# Assessment of hypokalemia and clinical prognosis in Patients with COVID-19 in Yangzhou, China

**DOI:** 10.1371/journal.pone.0271132

**Published:** 2022-07-08

**Authors:** Jiangtao Yin, Nana Yuan, Ziqiang Huang, Zhenkui Hu, Quanlei Bao, Zhenli Shao, Qiong Mei, Yong Xu, Wenli Wang, Dadong Liu, Li Zhao, Shengxia Wan

**Affiliations:** 1 Department of Critical Care Medicine, Affiliated Hospital of Jiangsu University, Zhenjiang, People’s Republic of China; 2 Institute of Digestive Diseases, Jiangsu University, Zhenjiang, People’s Republic of China; 3 Department of Infectious Diseases, The Third People’s Hospital of Yangzhou, Yangzhou, People’s Republic of China; 4 Department of Infectious Diseases, Affiliated Hospital of Jiangsu University, Zhenjiang, People’s Republic of China; 5 Nursing Department, Affiliated Hospital of Jiangsu University, Zhenjiang, People’s Republic of China; 6 Department of Critical Care Medicine, Jinling Hospital of Nanjing Medical University, Nanjing, People’s Republic of China; 7 Department of Endocrinology, Affiliated Hospital of Jiangsu University, Zhenjiang, People’s Republic of China; 8 Department of Neurology, The Fourth Affiliated Hospital of Jiangsu University, Zhenjiang, People’s Republic of China; GSVM Medical College, INDIA

## Abstract

**Background:**

Hypokalemia is a frequent electrolyte imbalance in patients with COVID-19. The aim of this study was to estimate the association between hypokalemia and clinical prognosis in patients with moderate COVID-19.

**Methods:**

A single-center, retrospective, observational study was conducted on 81 non-ICU admitted patients with moderate COVID-19 according to the criteria issued by the Chinese Health Bureau in the Third People’s Hospital of Yangzhou (Northern Jiangsu People’s Hospital New District Branch) from 4th to 25th August 2021. The demographic, clinical, and laboratory data were reviewed and collected, then the correlation between hypokalemia and prognosis was determined.

**Results:**

The level of serum potassium of patients ranged from 2.80 mmol/L to 4.70 mmol/L. Hypokalemia was detected in 39 out of the 81 included patients (48.15%) during hospitalization. Patients with hypokalemia had prolonged days of negative nucleic acid conversion and hospital stay. Correlation analysis showed that the level of serum potassium was negatively correlated with days of negative nucleic acid conversion and length of hospital stay. Bivariate logistic regression analysis proved that hypokalemia was a risk factor for prolonged hospital stay in patients with moderate COVID-19.

**Conclusion:**

Hypokalemia was prevalent in patients with moderate COVID-19 in Yangzhou, China. Hypokalemia was associated with the prolonged hospital stay in patients with moderate COVID-19.

## Introduction

COVID-19 is an emerging global pandemic emerged in December 2019 which mainly manifested by fever, dry cough, fatigue, etc [1, 2]. Epidemiological studies revealed that severe cases usually develop dyspnea after 1 week, and rapidly progress to acute respiratory distress syndrome, septic shock, metabolic acidosis, as well as coagulation dysfunction and multiple organ failure [3, 4]. Systemic release of cytokines is thought to be one of the causes of organ dysfunction and, simultaneously, the major determinant of morbidity in these patients [5].

Early case reports described that hypokalemia is a frequent lab abnormality [6]. Gastrointestinal losses, reduced intake, tubular damage and hyper-activation of the renin–angiotensin–aldosterone system (RAAS) appeared to be responsible for hypokalemia [6]. Gaetano and his colleagues found that 41% of COVID-19 patients developed hypokalemia in their study, moreover, female sex and diuretic therapy were the risk factors for hypokalemia which was unrelated to ICU transfer and mortality [7]. Another study disclosed that continued renal excretion of potassium due to excessive degradation of angiotensin-converting enzyme 2 [8].

There are still lots of unclear questions about low serum potassiumin COVID-19, to further investigate the epidemiological characteristics of hypokalemia and its association with clinical outcomes in patients with COVID-19, here we designed this retrospective study.

## Methods

### Study population

Adult patients with a diagnosis of moderate COVID-19 were recruited in the Third People’s Hospital of Yangzhou from 4th to 25th August 2021. All patients were aged older than 18 years and received a diagnosis of COVID-19 according to the criteria issued by the National Health Bureau of the People’s Republic of China.

### Study design

A single-center, retrospective, observational study was conducted in the Third People’s Hospital of Yangzhou from 4th to 25th August 2021. This was a retrospective and non-interventional study, which did not require an ethics committee to conduct an ethics review and waived the need for signed informed consent of the participants. However, oral informed consent was obtained from all patients. The research was conducted according to the Strengthening the Reporting of Observational Studies in Epidemiology (STROBE) reporting guideline. The demographic, clinical, and laboratory data were reviewed and collected by the trained medical staff then the correlation between hypokalemia and prognosis was analyzed.

### Data extraction

The extracted data included age, sex, comorbidities, laboratory data at the first day after admission, and clinical outcomes. The comorbidities included hypertension, diabetes, and cardiopathy. The laboratory data included hematocrit, white blood cell (WBC), lymphocyte, neutrophil, platelet, alanine transaminase (ALT), aspartate aminotransferase (AST), creatine kinase-MB (CK-MB), blood glucose, concentration of sodion, chloridion and calciumion, blood urea nitrogen (BUN), creatinine, uric acid and cystatin. The clinical outcomes included the prevalence of hypokalemia, assessment of the relationship between hypokalemia and days of negative nucleic acid conversion and length of hospital stay, as well as the hypokalemia on the risk of ICU transfer.

### Definitions

#### Hypokalemia

Clinically, the levels of serum potassium were measured on plasma and by automatic biochemical analyzer. The normal serum potassium reference level fluctuated between 3.5 to 5.5 mmol/L. Hypokalemia was defined as a serum potassium value less than 3.5 mmol/L at any time during the first day after admission. In addition, hypokalemia was classified as mild (serum potassium value was 3.0–3.4 mmol/L), moderate (serum potassium value was 2.5–3.0 mmol/L), and severe (serum potassium value was less than 2.5 mmol/L).

Days of negative nucleic acid conversion: days from hospital admission to two consecutive negative nucleic acid results with an interval of more than 24 hours.

Category of the COVID-19:according to the criteria issued by the Chinese Health Bureau [9], mild cases involved mild clinical manifestations and no pneumonia; moderate cases involved respiratory symptoms and mild pneumonia; severe cases involved respiratory distress (30 breaths/min), oxygen saturation (93% at rest), or arterial partial pressure of oxygen–fraction of inspired oxygen less than or equal to 300 mmHg; and critically ill cases were those that met any of respiratory failure criteria and required mechanical ventilation, or those with shock or other organ failure that required intensive care unit care.

### Statistical analysis

Data were processed by using SPSS 20.0 software. Continuous variables were presented as mean (standard deviation, SD) and analyzed by the independent-samples T test. Categorical variables were presented as frequencies (percentages), and analyzed by chi-square tests. Pearson correlation method was used to evaluate the correlation between serum potassium and days of negative nucleic acid conversion and length of hospital stay. Bivariate logistic regression was used to explore the relationship between hypokalemia and clinical outcomes in patients with moderate COVID-19. Variables were selected for inclusion in the models based on statistical significance in the univariate analyses. A *P* < 0.05 was considered statistically significant.

## Results

### Patient inclusion

**Fig 1:**
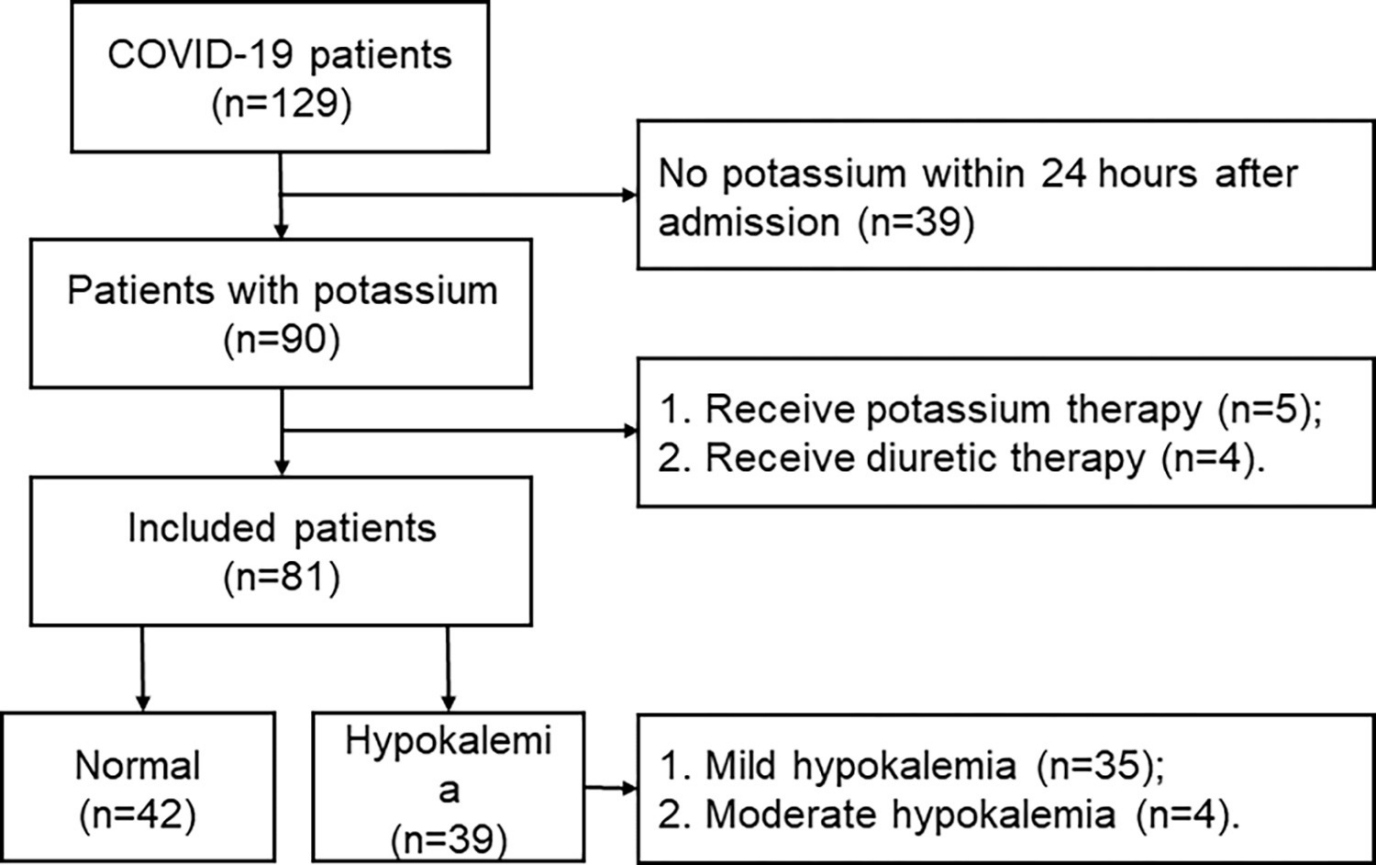
Flow diagram of the participants.

In total, 129 adult patients with moderate COVID-19 were screened in this study. After a rigorous screening process, 39 patients with no serum potassium test, 5 patients received potassium therapy and 4 patients received diuretic therapy were excluded from the study.Finally,81patientswere included in this study, 42 patients had the normal serum potassium level and 39 with hypokalemia, among the patients with hypokalemia, 4 patients had mild hypokalemia and 35 moderates (**Fig 1**). All of these included patients had one or more symptoms of COVID-19 (**S1 Table**). Among the included COVID-19 patients, 63 (77.78%) were vaccinated against COVID-19.

Demographics and clinical data of the included patients were reported in **Table 1**. Their mean age (SD) was 50.28 (15.68) years, and male accounted for 39.51%. The level of serum potassium ranged from 2.80 mmol/L to 4.70 mmol/L. Nearly half of them (48.15%) had mild to moderate hypokalemia. The majority of patients (89.74%) patients experienced only a mild decrease in serum potassium level. In addition, the hypokalemia patients had lower platelet counts, serum sodium and calcium levels, and higher AST level than those with normal serum potassium. All of the hypokalemia patient received oral potassium supplementation. After 3–5 days of treatment, blood potassium levels returned to normal in all hypokalemia patients.

**Table 1:** Data of the included patients with moderate COVID-19.

	Total (n = 81)	Hypokalemia (n = 39)	Normal (n = 42)	*P* value
Age (years)	50.28±15.68	53.59±15.60	47.21±16.91	0.067
Male (%)	32(39.51)	13(33.33)	19(45.24)	0.273
**Comorbidities(%)**				
Hypertension	25(30.86)	16(41.03)	9(21.43)	0.056
Diabetes	10(12.35)	4(10.26)	6(14.29)	0.582
Cardiopathy	7(8.64)	4(10.26)	3(7.14)	0.618
**Laboratory data at the first day after admission**				
Hematocrit (%)	39.42±4.83	39.27±4.55	39.56±5.12	0.796
WBC (10^9^/L)	5.81±2.07	5.59±2.43	6.01±1.67	0.357
Lymphocyte (10^9^/L)	1.11±0.44	1.14±0.51	1.08±0.36	0.516
Neutrophil (10^9^/L)	4.11±1.87	3.89±2.14	4.31±1.59	0.320
Platelet (10^9^/L)	191.21±61.17	175.23±56.13	206.05±62.56	0.023
ALT	23.81±16.58	26.00±17.35	21.78±15.78	0.255
AST	27.02±15.84	30.64±17.40	23.65±13.60	0.047
CK-MB	15.00±8.28	15.72±7.70	14.34±8.82	0.459
Blood glucose (mmol/L)	7.42±2.67	7.56±2.45	7.29±2.88	0.648
Sodion(mmol/L)	136.89±2.78	136.87±2.70	136.90±2.89	0.958
Chloridion(mmol/L)	98.84±3.35	97.97±3.43	99.64±3.10	0.024
Calcium ion(mmol/L)	2.31±0.09	2.29±0.09	2.33±0.09	0.040
BUN	4.76±2.65	4.55±2.72	4.95±2.60	0.502
Creatinine	75.32±26.94	73.21±27.51	77.29±26.59	0.499
Uric Acid	325.93±109.75	309.24±119.21	341.42±99.11	0.189
Cystatin	0.90±0.46	0.90±0.49	0.91±0.43	0.885
**Clinical outcomes**				
Days of negative nucleic acid conversion (days)	9.65±3.57	10.67±3.34	8.71±3.56	0.013
Length of hospital stay(days)	12.52±2.98	13.36±3.09	11.74±2.67	0.013
Risk of ICU transfer (%)	8(9.88)	4 (10.26)	4 (9.52)	0.942

WBC: white blood cell, ALT: alanine transaminase, AST: aspartate aminotransferase, CK-MB: creatine kinase-MB, BUN: blood urea nitrogen.

### Hypokalemia was a risk factor for poor prognosis

**Fig 2:**
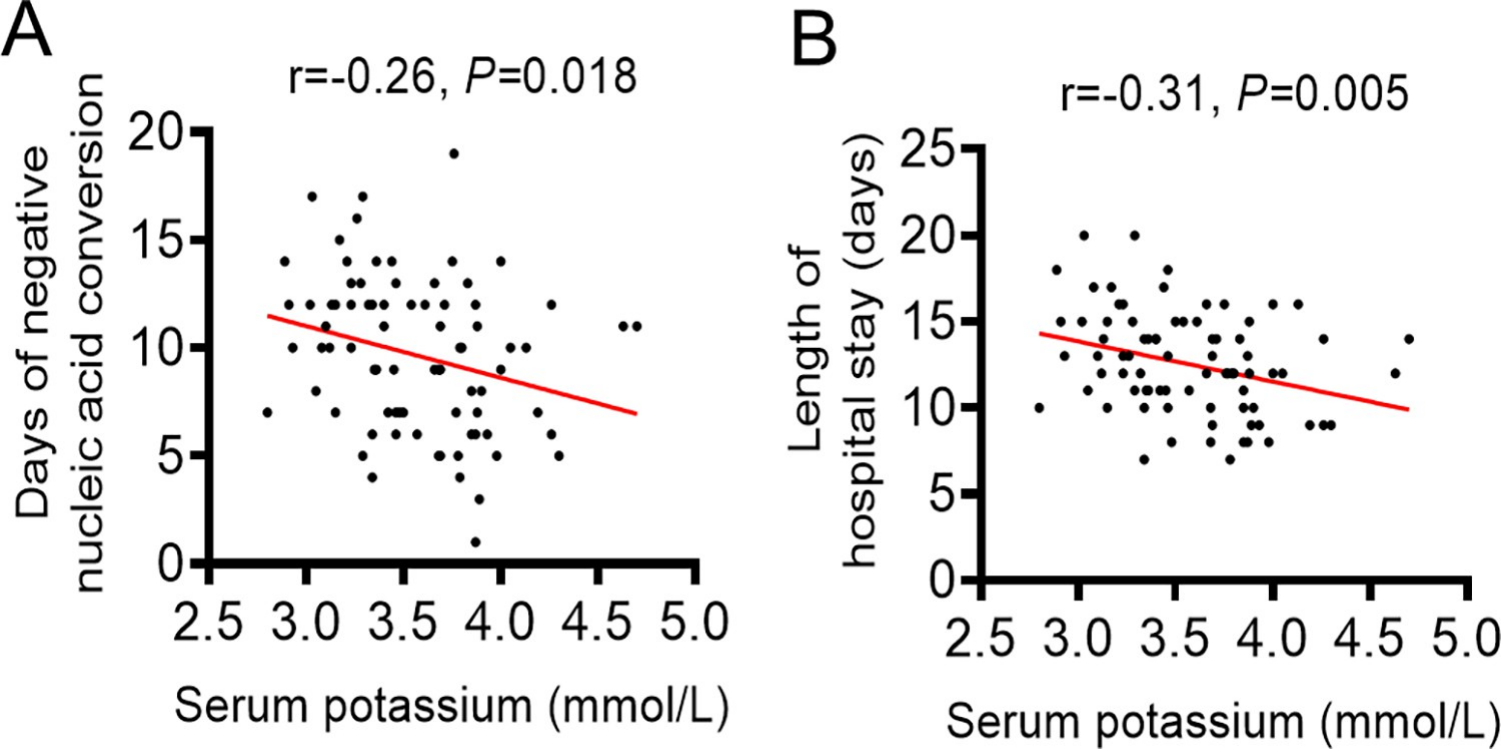
Correlation analysis showed that the level of serum potassium was negatively correlated with days of negative nucleic acid conversion (A) and length of hospital stay (B).

We found that days of negative nucleic acid conversion and length of hospital stay in patients with hypokalemia were significantly lengthened compared with those with normal serum potassium (**Table 1**). Furthermore, correlation analysis showed that the level of serum potassium was negatively correlated with days of negative nucleic acid conversion and length of hospital stay (**Fig 2**). We also found that the risk of ICU transfer in patients with hypokalemia was slightly higher than these with normal serum potassium, but the difference was not statistically significant. All of these indicated that hypokalemia was a risk factor for poor prognosis in patients with moderate COVID-19.

### Hypokalemia was not associated with prolonged days of negative nucleic acid conversion

According to whether the nucleic acid of the patients turned negative within 1 week, we divided the included patients into fast negative conversion (FNC) group (nucleic acid turned negative within 1 week) and slow negative conversion (SNC) group (nucleic acid turned negative longer than 1 week). Differences in basic data between the 2 groups were showed in **Table 2**. We found that compared with the SNC group, patients in the FNC group had a lower incidence of hypokalemia.

**Table 2:** Data of the included patients with moderate COVID-19 in FNC group and SNC group.

	Total (n = 81)	FNC group (n = 28)	SNC group (n = 53)	*P* value
Age (years)	50.28±15.68	44.96±15.53	53.09±15.16	0.026
Male (%)	32(39.51)	12(42.86)	20(37.74)	0.654
**Comorbidities (%)**				
Hypertension	25(30.86)	7(25.00)	18(33.96)	0.406
Diabetes	10(12.35)	3(10.71)	7(13.21)	0.746
Cardiopathy	7(8.64)	1(3.57)	6(11.32)	0.238
**Laboratory data at the first day after admission**				
Hematocrit (%)	39.42±4.83	40.38±4.39	38.91±5.01	0.195
WBC (10^9^/L)	5.81±2.07	6.52±2.22	5.43±1.91	0.024
Lymphocyte (10^9^/L)	1.11±0.44	1.29±0.52	1.02±0.36	0.007
Neutrophil (10^9^/L)	4.11±1.87	4.64±2.01	3.83±1.75	0.062
Platelet (10^9^/L)	191.21±61.17	208.71±62.59	181.96±15.23	0.061
ALT	23.81±16.58	23.18±19.17	24.15±15.23	0.804
AST	27.02±15.84	22.71±10.94	29.29±17.57	0.075
CK-MB	15.00±8.28	13.08±4.35	16.02±9.62	0.128
Blood glucose (mmol/L)	7.42±2.67	6.88±2.27	7.70±2.83	0.189
Hypokalemia (%)	39(48.15)	9(32.14)	30(56.60)	0.036
Sodion (mmol/L)	136.89±2.78	137.25±2.78	136.70±2.79	0.400
Chloridion (mmol/L)	98.84±3.35	99.39±3.28	98.55±3.38	0.282
Calcium ion (mmol/L)	2.31±0.09	2.34±0.08	2.29±0.09	0.008
BUN	4.76±2.65	4.40±2.32	4.95±2.81	0.380
Creatinine	75.32±26.94	70.43±22.47	77.91±28.90	0.237
Uric Acid	325.93±109.75	321.30±118.28	328.38±106.06	0.785
Cystatin	0.90±0.46	0.89±0.47	0.91±0.45	0.857

FNC: fast negative conversion, SNC: slow negative conversion, WBC: white blood cell, ALT: alanine transaminase, AST: aspartate aminotransferase, CK-MB: creatine kinase-MB, BUN: blood urea nitrogen.

To explore the effect of hypokalemia on the days of negative nucleic acid conversion, the normal potassium was selected as the reference group for the bivariate logistic regression models. The results demonstrated that hypokalemia was not associated with prolonged days of negative nucleic acid conversion among moderate COVID-19 patients with or without adjusted variables (**S2 Table**).

### Hypokalemia was a risk factor for prolonged hospital stay

The mean hospitalization time of the included patients was 12.52 days. Accordingly, the patients were divided into short-term hospitalization (STH) group (length of hospital stay< 12.52 days) and long-term hospitalization (LTH) group (length of hospital stay> 12.52 days). Differences in basic data between the 2 groups were reported in **Table 3**. We found that patients in the STH group had a low incidence of hypokalemia. Meanwhile, the patients with hypokalemia had a high level of HCT.

**Table 3:** Data of the included patients with moderate COVID-19 in STH group and LTH group.

	Total (n = 81)	STH (n = 43)	LTH (n = 38)	*P* value
Age (years)	50.28±15.68	47.21±15.19	53.76±15.70	0.060
Male (%)	32(39.51)	21(48.84)	11(28.95)	0.068
**Comorbidities (%)**				
Hypertension	25(30.86)	12(27.91)	13(34.21)	0.540
Diabetes	10(12.35)	6(13.95)	4(10.53)	0.640
Cardiopathy	7(8.64)	2(4.65)	5(13.16)	0.174
**Laboratory data at the first day after admission**				
Hematocrit (%)	39.42±4.83	40.65±4.27	38.03±5.09	0.014
WBC (10^9^/L)	5.81±2.07	6.22±2.02	5.34±2.06	0.057
Lymphocyte (10^9^/L)	1.11±0.44	1.16±0.48	1.05±0.38	0.249
Neutrophil (10^9^/L)	4.11±1.87	4.48±1.86	3.69±1.83	0.060
Platelet (10^9^/L)	191.21±61.17	201.58±65.45	179.47±54.42	0.105
ALT	23.81±16.58	24.50±17.78	23.04±15.32	0.695
AST	27.02±15.84	25.60±15.21	28.63±16.58	0.394
CK-MB	15.00±8.28	13.78±7.35	16.39±9.12	0.157
Blood glucose (mmol/L)	7.42±2.67	7.31±2.46	7.55±2.91	0.687
Hypokalemia (%)	39(48.15)	16(37.21)	23(60.5)	0.036
Sodion (mmol/L)	136.89±2.78	137.23±3.24	136.50±2.14	0.240
Chloridion (mmol/L)	98.84±3.35	99.33±3.42	98.29±3.22	0.166
Calcium ion (mmol/L)	2.31±0.09	2.32±0.09	2.29±0.09	0.224
BUN	4.76±2.65	4.58±2.48	4.96±2.85	0.526
Creatinine	75.32±26.94	73.74±24.39	77.11±29.80	0.579
Uric Acid	325.93±109.75	326.97±99.81	324.75±121.38	0.928
Cystatin	0.90±0.46	0.88±0.42	0.93±0.50	0.630

STH: short-term hospitalization, LTH: long-term hospitalization, WBC: white blood cell, ALT: alanine transaminase, AST: aspartate aminotransferase, CK-MB: creatine kinase-MB, BUN: blood urea nitrogen.

To explore the effect of hypokalemia on hospitalization time, the normal potassium was selected as the reference group for the bivariate logistic regression models. The results showed that hypokalemia was associated with prolonged hospital stay among moderate COVID-19 patients with or without adjusted hematocrit (**S3 Table**).

## Discussion

We found that many patients with COVID-19 experienced hypokalemia clinically, therefore, we designed this retrospective clinical study so as to elucidate the prevalence of hypokalemia and its association with clinical outcomes.

In our study, 48.15% of patients with moderate COVID-19 suffered from hypokalemia which was consistent with previous findings [7]. To clarify the relationship between hypokalemia and clinical outcomes, we chosed days of negative nucleic acid conversion, length of hospital stay and risk of ICU transfer as the observation indicators, and we first discovered that patients with hypokalemia had prolonged days of negative nucleic acid conversion and hospital stay relative to those with normokalemia. Moreover, our study revealed that the level of serum potassium was negatively correlated with days of negative nucleic acid conversion and length of hospital stay. Further research found that hypokalemia was associated with prolonged hospital stay in patients with moderate COVID-19. Negative nucleic acid means the disruption of replication of SARS-CoV-2, and two consecutive negative nucleic acid tests with an interval of more than 24 hours are one of the discharge criteria according to the National Health Bureau, so we chosed it as one of the primary clinical outcomes.

Few data are available on serum potassium level in patients with COVID-19 due to the emerging infectious disease. Potassium is the main cation which maintains the physiological activities of cells, and plays an important role in maintaining the normal osmotic pressure and acid-base balance of the body, participating in sugar and protein metabolism, as well as ensuring the normal function of neuromuscular [10, 11]. Hypokalemia occurs in many different conditions, such as decreased intake, intracellular transfers of serum potassium, excessive renal and gastrointestinal losses, etc [12]. Usually, hypokalemia is defined when the level of serum potassium is less than 3.5mmol/L [13]. The primary symptoms of patients include fatigue, muscle weakness, abdominal distension and arrhythmia, etc. The severity of hypokalemia depends on the degree of intracellular and extracellular potassium deficiency and the rate at which potassium deficiency occurs. The symptoms of acute hypokalemia are more severe than chronic hypokalemia with the same level of potassium deficiency, and multiple organs of the body will be affected, including the cardiovascular, urinary, and neuromuscular system [14, 15].

Several reasons of potassium deficiency are involved in patients with COVID-19. On the one hand, poor appetite and diarrhea in some patients lead to hypokalemia. On the other hand, increased urinary K^+^ output also result to potassium deficiency. It is well known that the activity of rennin-angiotensin system (RAS) is balanced by angiotensin-converting enzyme inhibitors (ACEI, which increases RAS activity) and angiotensin-converting enzyme 2 (ACE2, which decreases RAS activity) [16]. Previous studies have shown that SARS-CoV-2 and SARS-CoV utilize the same host cell ACE2 as their cell entry receptor [17]. When SARS-CoV-2binds and degrades ACE2, the ability of ACE2 to regulate RAS is decreased and it cannot fully antagonize ACEI [18]. Finally, the RAS activity is increased which stimulates the adrenal cortex to secrete aldosterone, then enhances the distal delivery of sodium and water to collecting tubule of the kidney and the excretion of potassium [18]. Hypokalemia causes impaired reabsorption of chloride ions, eventually leading to hypochloremic alkalosis which was consistent with our observations [7]. In our study, we also found that patients with hypokalemia had worse clinical outcomes including prolonged days of negative nucleic acid conversion and hospital stay which indicated that reasonable correction of hypokalemia might improve patient prognosis. Interestingly, the hypokalemia prevalence in East Asian and European populations are different, and primary due to the East Asian populations have much higher ACE2 expression in tissues, which may suggest the variable susceptibility or response to SARS-CoV-2 of different populations under similar conditions [19, 20].

Lymphocyte count indicates immune function of the body. Usually, in viral infections, people with powerful immune systems possess greater ability to clear the virus. In this research, the lymphocyte count in patients whose nucleic acid converted negative faster was significantly higher than patients of slow negative conversion, which indicated that the former had stronger immune function, and provided an idea for the treatment of COVID-19. Oscar reported that hypokalemia was an independent predictor of IMV requirement and seemed to be a sensitive biomarker of severe progression of COVID-19 [6]. Another research about hypokalemia and COVID-19 documented that female sex and diuretic therapy were identified as risk factors for low serum potassium levels, but hypokalemia was unrelated to ICU transfer and death in that cohort of patients [7]. Notely, Our data showed that hypokalemia was associated with prolonged hospital stay with or without adjusted hematocrit.

There are also some limitations in our study. First, this is an observational, retrospective, single-center study, and collection of data was not standardized in advance. Second, results may be biased due to the small sample size. Third, the absence of data about arterial blood gas analysis and treatment, hampers interpreting our results.

## Conclusions

The present study identified that hypokalemia was a frequent electrolyte disorder in patients with moderate COVID-19. Patients with hypokalemia were more prone to have prolonged days of negative nucleic acid conversion and hospital stay. The level of serum potassium was negatively correlated with days of negative nucleic acid conversion and length of hospital stay. Finally, hypokalemia was associated with prolonged hospital stay in patients with moderate COVID-19.

## Supporting information

S1 Table Clinical Symptoms of the included patients (%). (DOCX)

S2 Table Hypokalemia was not associated with prolonged days of negative nucleic acid conversion between FNC group and SNC group. **Model 1:** Unadjusted model. **Model 2:** Adjusted for age. **Model 3:** Adjusted for variables included in model 2 + WBC, lymphocyte, and serum calcium ion.
(DOCX)

S3 Table Hypokalemia was associated with prolonged hospital stay. **Model 1:** Unadjusted model. **Model 2:** Adjusted for hematocrit.
(DOCX)
