# Supplementary material for: Assessment of hypokalemia and clinical prognosis in Patients with COVID-19 in Yangzhou, China
Source: PLoS One. 2022 Jul 8;17(7):e0271132. doi: 10.1371/journal.pone.0271132 (PMC9269409; doi:10.1371/journal.pone.0271132)
Supplement: S1 Table — (DOCX) [file pone.0271132.s001.docx]

**S1 Table. Clinical Symptoms of the included Patients (%).**

|  | **Total**  **(n=81)** | **Hypokalemia（n=39）** | **Normal（n=42）** | ***P* value** |
| --- | --- | --- | --- | --- |
| Fever (>37 °C) | 33(40.74) | 19(48.27) | 14(33.33) | 0.159 |
| Dry cough | 39(48.15) | 19(48.27) | 20(47.62) | 0.921 |
| Runny nose | 9(11.11) | 4(10.26) | 5(11.90) | 0.814 |
| Sore throat | 13(16.05) | 7(17.95) | 6(14.29) | 0.654 |
| Diarrhea | 3(3.70) | 0(0.00) | 3(7.14) | 0.089 |
| Faintness | 10(12.35) | 5(12.82) | 5(11.90) | 0.900 |
| Stuffy nose | 15(18.52) | 9(23.08) | 6(14.29) | 0.309 |
| Decreased sense of smell and taste | 6(7.41) | 2(5.13) | 4(9.52) | 0.450 |
| Myalgia | 5(6.17) | 3(7.69) | 2(4.76) | 0.584 |
| Conjunctivitis | 1(1.23) | 0(0.00) | 1(2.38) | 0.332 |
