# Supplementary material for: Assessment of hypokalemia and clinical prognosis in Patients with COVID-19 in Yangzhou, China
Source: PLoS One. 2022 Jul 8;17(7):e0271132. doi: 10.1371/journal.pone.0271132 (PMC9269409; doi:10.1371/journal.pone.0271132)
Supplement: S2 Table — Model 1: Unadjusted model. Model 2: Adjusted for age. Model 3: Adjusted for variables included in model 2 + WBC, lymphocyte, and serum calcium ion. (DOCX) [file pone.0271132.s002.docx]

**S2 Table.** **Hypokalemia was not associated with prolonged** **days of negative nucleic acid conversion between FNC group and SNC group.**

| **Models** | **OR** | **95% CI** | ***P* value** |
| --- | --- | --- | --- |
| **Model 1** |  |  |  |
| Hypokalemia | 2.18 | 0.85, 5.59 | 0.106 |
| **Model 2** |  |  |  |
| Hypokalemia | 1.82 | 0.68, 4.84 | 0.231 |
| **Model 3** |  |  |  |
| Hypokalemia | 1.69 | 0.56, 5.11 | 0.349 |

**Model 1:** Unadjusted model.

**Model 2:** Adjusted for age.

**Model 3:** Adjusted for variables included in model 2 + WBC, lymphocyte, and serum calcium ion.
