# Supplementary material for: Assessment of hypokalemia and clinical prognosis in Patients with COVID-19 in Yangzhou, China
Source: PLoS One. 2022 Jul 8;17(7):e0271132. doi: 10.1371/journal.pone.0271132 (PMC9269409; doi:10.1371/journal.pone.0271132)
Supplement: S3 Table — Model 1: Unadjusted model. Model 2: Adjusted for hematocrit. (DOCX) [file pone.0271132.s003.docx]

**S3 Table.** **Hypokalemia was associated with prolonged hospital stay.**

| **Models** | **OR** | **95% CI** | ***P* value** |
| --- | --- | --- | --- |
| **Model 1** |  |  |  |
| Hypokalemia | 2.59 | 1.06, 6.35 | 0.038 |
| **Model 2** |  |  |  |
| Hypokalemia | 2.70 | 1.06, 6.89 | 0.038 |

**Model 1:** Unadjusted model.

**Model 2:** Adjusted for hematocrit.
